# Sociodemographic characteristics of professional categories most affected by COVID-19 in Mozambique from March to July 2020

**DOI:** 10.11604/pamj.2022.43.155.30572

**Published:** 2022-11-24

**Authors:** Fabião Edmundo Maússe, Gerson Afai, Auria Ribeiro Banze, Judite Monteiro Braga, Tatiana Marrufo, Erika Valeska Rossetto, Cynthia Semá Baltazar

**Affiliations:** 1 Field Epidemiology and Laboratory Training Program, National Institute of Health, Maputo, Mozambique,; 2 Nacional Institute of Health, Maputo, Mozambique,; 3 Mass Genics Assigned to Mozambique, Centers for Disease Control and Prevention, Maputo, Mozambique

**Keywords:** COVID-19, epidemiology, public health surveillance, Mozambique

## Abstract

**Introduction:**

the risk of a worker becoming ill due to coronavirus disease 2019 (COVID-19) is related to occupational exposure to severe acute respiratory syndrome coronavirus 2 (SARS-CoV-2). Due to the need to restore work activities in Mozambique, the study was conducted with the aim of identifying the occupational categories most affected by COVID-19 in the former in the period from March to July 2020.

**Methods:**

this is a cross-sectional descriptive study, in which data from professions of confirmed cases of COVID-19 from 22 March to 29 July 2020 in Mozambique were analyzed. The professionals' data were reported daily by the National Institute of Health (NIH) and merged into a single database and exported to Excel, the latter categorized according to standard operating procedure (SOP) and descriptive statistics performed for its analysis.

**Results:**

in the period under analysis, 1,127 professionals were diagnosed with COVID-19, divided into 11 categories. Nampula province had the highest frequency of cases with 25.00% (277). The highest frequency of cases was registered in the domestic professional category, which had 16.77% (189/1,127) with the female sex being more frequent, 79.37% (150/189); and defense and security had 14.20% (160/1,127) of cases and male gender with 91.25% (146/160); Health workers had 13.04% (147/1,127), and the maximum number of COVID-19 cases was recorded in June with 58.50% (86/147).

**Conclusion:**

the professional categories most affected by COVID-19 in the period under review correspond to those groups that carry out activities requiring a physical presence at the workplace and from this; it is recommended that professionals reinforce preventive measures.

## Introduction

Since its appearance in Wuhan, China in December 2019, the severe acute respiratory syndrome coronavirus 2 (SARS-CoV-2) had infected until July 31^st^, 2020 17,106,007 people worldwide [1,2]. The risk of a person contracting coronavirus disease 2019 (COVID-19) may be associated with the type of work activity and occupational exposure. The risk may be low for workers without frequent close contact with others and workers providing teleservices, medium for people working in crowds or in contact with people returning from regions with high COVID-19 transmission, and high among those working on the frontline for the COVID-19 response, ambulance drivers carrying patients with COVID-19 and laboratory technicians [3,4]. A study in England showed that the risk of contracting COVID-19 was seven times higher in health care workers, and twice as high for social care workers and public transport workers compared with workers in non-essential services [5]. Occupational risks refer to the probability of injury or illness occurring within the workplace, which may include biological, physical and psychosocial risks. Pandemic COVID-19 introduced new occupational risks, which vary substantially between occupations [6]. Occupational risk for exposure to COVID-19 can be classified into very high, high, medium, and low. Very high risk is considered to be jobs with a high potential for exposure to known or suspected sources of COVID-19 during specific medical, postmortem or laboratory procedures. Healthcare and morgue workers are considered to be in this category [3]. Jobs with a high potential for exposure to known or suspected sources of COVID-19 are considered high-risk occupations. Healthcare providers, healthcare support workers, medical transport and mortuary workers exposed to bodies of people known or suspected to have COVID-19 at the time of death are considered in this class [3]. The objectives of the study were: i) to identify the professional categories most affected by COVID-19, in the period March to July 2020; ii) to describe the group of health professionals affected by COVID-19, from March to July 2020.

For the medium risk of exposure, work requiring frequent/close contact with people who may be infected but who are not known or suspected to have COVID-19 is considered. This class includes workers who have contact with the general public, including individuals returning from places with widespread transmission of COVID-19 [3]. Low risk of exposure includes work that does not require contact with known or suspected infected persons. In this category are considered those professionals who have minimal contact with the public and other co-workers [3]. In Mozambique the first case of COVID-19 was registered on March 22^nd^, 2020, and on April 1^st^ the State of Emergency was declared, limiting the movement of people in order to prevent and limit the transmission of COVID-19 until 31^st^ July 2020 [7,8]. According to SARS-CoV-2 sero-epidemiological surveys conducted in the cities of Nampula and Maputo, market vendors had the highest positivity rate, with 10.0% and 5.2%, respectively, just like the surveys conducted in other provinces such as Inhambane, Tete, Chimoio and Sofala. In the aforementioned surveys, among health professionals, the positivity rate was 7.0% in Nampula and 2.6% in Maputo [9,10].

## Methods

**Study design and population:** this is a cross-sectional descriptive study that included professional categories of COVID-19 cases reported in the period 22^nd^ March to 29^th^ July 2020. During the study period, 1,127 professionals from the 11 provinces of Mozambique of both genders who tested positive for COVID-19 were included.

**Study setting:** the study was conducted in Mozambique which is situated on the east coast of Africa. It is bordered by Swaziland to the south, South Africa to the southwest, Zimbabwe to the west, Zambia and Malawi to the Northeast, Tanzania to the North and the Indian Ocean to the East. It has an area of 799,380 km^2^, a coastline of almost 2,700 km from north to south and a population estimated at 27,909,798 according to the latest census [11].

**Data collection:** the data were extracted from daily databases of positive cases of COVID-19 from the National Health Observatory (NHO) of NHI. A standard operating procedure (SOP) was created to standardize the group of professions according to the professional classification in Mozambique [12]. For professions that did not fit into the professional categories already classified, the category “other areas” was created, which included gardener, timekeeper, supervisor, fisherman, enumerator, higher technician. The records that presented the occupation filled in as “minor/minors” were excluded from the analysis. These cases presented ages ranging from zero to 17 years.

**Availability of data and materials:** the data sets generated and analyzed during the current study are available from the corresponding author on reasonable request.

### Data analysis

**Descriptive epidemiology:** the cases reported daily from NIH were merged into a single database and exported to Excel for descriptive analysis. The daily reported cases from the NIH were merged into a single database and exported to Excel from where subsequent descriptive analyzes (frequencies) were performed. For the descriptive analysis the cases the professionals were described in person, time in place. For the analysis of professions, an aggregation of the data of daily cases in a single database and a subsequent data cleaning in an Excel spreadsheet was performed, which consisted of: correction and standardization of the names of professions, and creation of a new variable with the standardized professional categorization.

## Results

**Figure 1:**
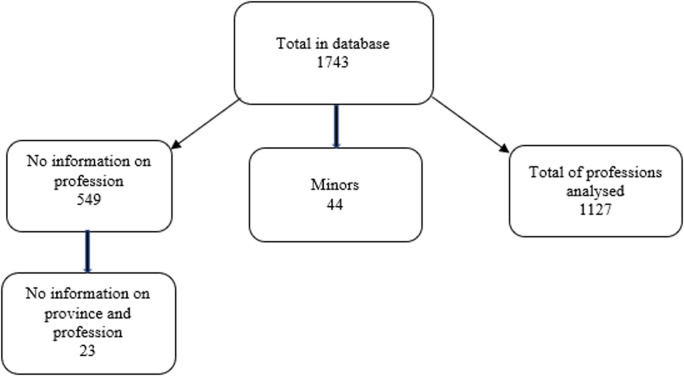
flowchart of COVID-19 case selection for occupational category analysis, March to July 2020

**Figure 2:**
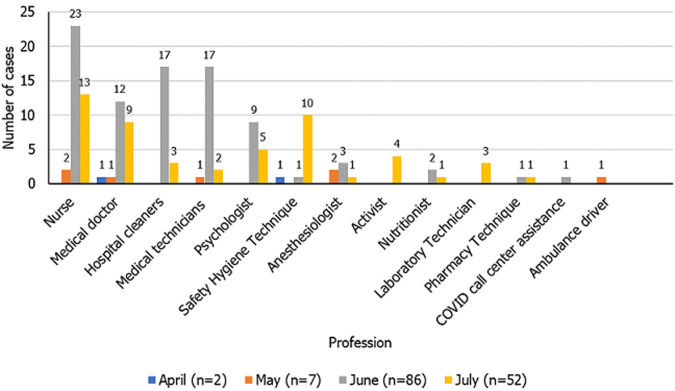
distribution of COVID-19 cases by occupational categories, Mozambique, March-July 2020

**Description of most affected occupational category:** after cleaning for inconsistency and duplications, the database was left with a total of 1743 registered cases, of which 549 cases without information of profession were excluded, and 23 cases without information of both profession and province. Also excluded were 44 individuals aged 0 to 17 years, who in the profession variable were registered as minors, and for the final analysis 1127 cases were based (Figure 1). During the surveillance period, 11 occupation categories were identified among the 1,127 cases analyzed (Table 1). The highest number of cases was recorded in the domestic category with 16.77% (189/1,127), followed by defense and security with, 14.20% (160/1,127), and health workers, and students with 13.04% (147/1,127) each (Figure 2). The median age of all cases was 32 (range 5-89) years (IQR=25;42), with more cases in the 30-39 group age (29.37%; 331/1,127), and most (60.96%; 687/1,127) of cases were male. The median age of cases in the domestic category was 36 (range: 18-84) years old. The largest proportion of cases in this category were in the 20-29 age group (32.28%; 61/189), and the majority (79.37%; 150/189) were female. The largest proportion (61.90%; 117/189) of cases in this category were reported in July, followed by June with 35.98% (68/189), and Maputo province reported the greatest number, with 23.28% (44/189), followed by Nampula and Cabo Delgado with 19.0% (36/189) and 17.46% (33/189), respectively. The cases in the defense and security category had a median age of 30 years, with a range of 20-84 year, and the large majority (91.25%; 146/160) of the cases were male. The greatest proportion of the cases was in the 20-29 age group (47.50%; 76/160). Most cases were reported in July (61.50%; 98/160), followed by June, with 31.63% (49/160). Maputo province had the highest number of cases in this category with 33.13% (53/160), followed by Cabo Delgado with 30.63% (49/160), and Maputo City and Nampula, with 12.50% (20/160) each province. The median age of the student category was 17 years, ranged 5-66 years, with approximately half (51.02%; 75/147) of the cases reported in males and in the 10-19 age group (52.38%; 77/147). More cases reported in the student category were in June and July, with 45.58% (67/147) and 48.30% (71/147) respectively. Among students, the provinces with the most cases were Nampula (29.25%; 43/147), Cabo Delgado (22.45%; 33/147), and Maputo Province (17.01%; 25/147).

**Table 1:** distribution of COVID-19 cases by sociodemographic characteristics and occupational categories (N=1.127), Mozambique, March to July 2020

Characteristic	n	%
**Gender**		
Male	684	61.0
Female	443	39.0
**Age group**		
5-9	98	8.7
10-19	326	28.9
20-29	331	29.4
30-39	175	15.5
40-49	91	8.1
50-59	72	6.4
>60	34	3.0
**Province**		
Nampula	277	24.58
Cabo Delgado	259	22.98
Maputo Province	236	20.94
Maputo Cidade	173	15.35
Gaza	36	3.19
Inhambane	31	2.75
Tete	31	2.75
Zambezia	29	2.57
Sofala	21	1.86
Niassa	18	1.60
Manica	16	1.42
**Professional category**		
Domestic	189	16.77
Security and defense	160	14.20
Student	147	13.04
Health worker	147	13.04
Administration and management	132	11.71
Buckberry/hotel	123	10.91
Transport	94	8.34
Engineer	69	6.12
Other areas	33	2.93
Agriculture/livestock	23	2.04%
Unemployed	10	0.89%

**Figure 3:**
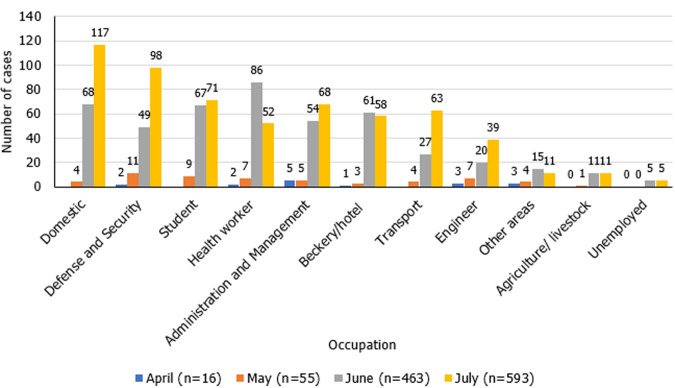
distribution of COVID-19 cases in the health category, Mozambique, March-July 2020

**Health care occupational category:** the health care workers category had a median age of 34 (19-89) years, with 53.74% (79/147) of cases reported among females. The largest proportion of cases (44.22%; 65/147) was in the 30-39 age group. Among the Health workers, nurses represented 25.85% (38/147), followed by medical doctors, with 14.97% (22/147). Hospital cleaners and medical technicians each registered 13.61% (20/147) of COVID-19 cases in health care workers. Over half (58.50%; 86/147) of health care workers tested positive for COVID-19 in June, with nurses registering 26.74% (23/86) of cases, followed by medical technicians and hospital cleaners, both with 19.77% (17/86) of cases, and lastly medical doctors (13.95%; 12/86) (Figure 3). In July, COVID-19 cases decreased to 35.37% (52/147), though nursing remained the profession with the greatest number of cases, with 25.00% (13/52). The most health workers with COVID-19 were from Nampula province (51.02%; 75/147), and the provinces of Cabo Delgado and Maputo City were in third, with 20.41% (30/147) and 11.56% (17/147) cases, respectively. Between March and July 29, three pregnant women were identified with COVID-19, one aged 24 and two aged 28. These cases were in the Health Worker, Student and Financial occupational categories. The symptoms were mild, with two of the three women describing headache and cough. All three were diagnosed in July; two were from Cabo Delgado province, and one was from Maputo City.

## Discussion

Between March 22^nd^ and July 29^th^, 2020, the domestic category had the highest number of cases. This occupation comprised of people who provide services to the homes of others, and people with this occupation may have made up the largest number of cases potentially due to their tendency to move from their homes to the workplaces daily on overcrowded public transportation. During the state of emergency these workers also may not have been dismissed by employers and thus may have had increased risk of exposure to SARS-CoV-2 during the study period. Domestic workers, as an occupation category that moves between homes and different family groups, can be a source of transmission and maintain a large transmission chain if they do not comply with prevention measures and quarantine properly [5]. The high number of cases differs from the sero-epidemiological studies conducted in Nampula and Maputo City in which found the market vendors to have the greatest number of cases [9,10]. Defense and Security professionals (military), due to the public nature of their activities, may not be able to maintain all preventive measures, such as changing their masks periodically and washing their hands frequently. The military may be a group at particular risk of respiratory diseases due to housing in barracks, transport during missions, and inadequate facilities for hygiene measures which can pose a risk for coronavirus infection in military personnel [13,14]. In Mozambique, studies on susceptibility of military personnel to respiratory infections are non-existent, making this a challenge for researchers. The high number of cases among students, may be a consequence of non-compliance with social distancing measures or they also may have been exposed by family members working outside the home. The non-compliance suggests two possible situations: social gatherings or work activities to supplement income during the state of emergency [15,16]. School closures are a measure to prevent COVID-19 by distancing students from schools, which has contributed to the decrease of COVID-19 cases [17]. In Mozambique, until July, the number of students with COVID-19 was tending to increase.

In health care workers, we found that women, and the 31-40 age group had the highest number of cases, which can reflect the demographic characteristics of the health care workforce in Mozambique. Studies conducted in Brazil and the United States of America (USA) found that the majority of health professionals testing positive for COVID-19 were female; however, implies discordance as the predominant age group was 40-49 in Brazil and 16-44 years in the USA [18,19]. In this study, nurses had the highest number of cases in the health worker category, possibly due to be in a frontline COVID-19 group that have more contact with COVID-19 patients and exposures for a longer period as compared to other health professions [5]. These findings corroborate the previous study in Brazil that found nurses as representing the largest number of COVID-19 cases among health care workers [19]. The number of health workers who tested positive for COVID-19 increased in the month of June and then decreased in July. The decrease in infections among health care workers, in a period in which the number of infections was increasing, may be the result of an improvement in awareness in taking preventive measures or due to the correct use and availability of personal protection equipment, and the dissemination of COVID-19 prevention norms [17]. Although a small number of pregnant women were infected to COVID-19 in this study, prevention and treatment strategies should be considered because pregnant women are more susceptible to respiratory pathogens due to hormonal changes and a weakened immune system [20]. Although there is a lack of information regarding the transmission of SARS-CoV-2 from mother to fetus, studies have shown that in pregnant women with COVID-19, there may be obstetric complications such as pre-eclampsia, premature rupture of the membrane, and irregular uterine contractions, leading to premature birth [20-22]. The study had some limitations that may have influenced the sample size such as the inconsistency of the total number of cases, in some dates, between the database and in the daily bulletins of COVID-19 of INS and the incompleteness in the recording of variables such as province, profession and age in some positive cases.

## Conclusion

The professional categories most affected by COVID-19 in the analyzed period correspond to the groups that perform activities that require a physical presence in the workplace, and can be due to social vulnerability as financial status, to perform what leads to perform activities in different locations. It is recommended to strengthen the public health prevention measures targeting professional categories most affected such as health professionals as an area that works directly with patients.

### What is known about this topic

- People who work at home and without contact with the outside environment are less susceptible to SARS-CoV-2 infection;
- People working on the first line for COVID-19 are more susceptible to SARS-CoV-2 infection.

### What this study adds

- Domestic workers are more prone to COVID-19 because of multiply exposures in different places;
- Military should consider scaling up implementation of protective measures against COVID-19, especially the use of personal protective equipment.
